# Role of Stress Myocardial Scintigraphy in the Evaluation of Incompletely Revascularized Post-PCI Patients

**DOI:** 10.1155/2011/180936

**Published:** 2011-09-21

**Authors:** Alfredo R. Galassi, Francesco Marzá, Salvatore Azzarelli, Salvatore D. Tomasello

**Affiliations:** ^1^Department of Internal Medicine and Systemic Disease, Clinical Division of Cardiology, Ferrarotto Hospital, University of Catania, Via Antonello da Messina 75 Acicastello, 95021 Catania, Italy; ^2^Division of Cardiology, Cannizzaro Hospital, Via Messina 829, 95126 Catania, Italy

## Abstract

Percutaneous coronary intervention (PCI) is actually the most used method of revascularization. Although complete revascularization remains a desirable goal, it may not be possible or not easy to plan in many patients. Thus, incomplete revascularization might be a preferred treatment strategy in selected patient categories. Stress myocardial scintigraphy, because of its high diagnostic accuracy and prognostic value and its ability to assess location and extent of myocardial ischemia regardless of symptoms as well as to evaluate patients who are unable to exercise or who have uninterpretable electrocardiogram, is of paramount importance for clinical decision making in patients with multivessel disease and incomplete revascularization.

## 1. Introduction

For many patients with multivessel disease, PCI is a definitive alternative to coronary artery by-pass graft (CABG) [1, 2]. Complete revascularization remains a desirable goal, and a satisfactory outcome may be obtained with complete revascularization with PTCA [3] or CABG [4, 5].

However, complete revascularization may not be possible in many patients, either because of the operator inability to treat all diseased coronary arteries such as chronic total occlusions and adverse stenosis morphology or simply being not easy to plan in the presence of coronary narrowing less than severe (>50% but <70%). Alternatively, the operator might decide to selectively revascularize only large areas of myocardium at risk. Indeed, clinical reasons for this decision include, among others, unstable angina, recent myocardial infarction, severe left ventricular dysfunction, urgent/emergent PCI, preexisting renal failure for which the amount of angiographic contrast media should be limited, and premature termination of the procedure for unexpected problems, such as in those elderly or frail patients who cannot lay flat for prolonged periods. Thus, revascularization of the culprit lesion may be part of an incomplete revascularization strategy which may be of value other than a common clinical practice in many selected cases, when wisely chosen because of its easier achievement and lower immediate risk.

## 2. Historical and Updated Background of Anatomical Incomplete versus Anatomical Complete Revascularization

In the BARI Trial comparing CABG with multivessel PCI, the 10-year survival did not differ between the two treatments despite the fact that 91% of significant lesions were bypassed in the CABG patients compared to 54% of significant lesions in the PCI patients [4]. In the same trial, cardiac death, myocardial infarction and angina at 10-year follow-up were similar in patients in whom a PCI was performed as a planned incomplete revascularization, as compared to those with a planned complete revascularization. Furthermore, even in patients for whom complete revascularization with angioplasty was planned, only 50% of lesions were both attempted and dilated. Similar results were obtained by Vandormael et al. who found an important symptomatic relief achieved with partial revascularization in patients with multivessel disease. In these patients, 1-year cardiac event rate was not significantly higher than the rate in the group with complete revascularization. At followup, 66% of patients with complete revascularization were asymptomatic and 84% had clinical improvement; this was similar to 58% and 85%, respectively, for patients with incomplete revascularization [10]. Thus, incomplete revascularization seems to be a valuable solution when a culprit lesion can be identified, particularly when this vessel is a favourable lesion which serves a large noninfarct territory, or in case of an acute coronary syndrome where there is the need to stabilize patient's condition [11, 12]. Moreover, in the Arterial Revascularization Therapies Studies (ARTS), randomization of stenting in patients with incomplete revascularization including diabetics did not influence late mortality [13]. 

However, in multivessel disease, complete revascularization is often an achievable goal not often pursued both by PCI and CABG, despite the fact that the latter is generally more complete. Indeed, even when complete revascularization is planned such as in the chronic total occlusion subgroup of the Syntax Trial, complete revascularization is obtained in only 49.4% of PCI treated patients while in only 68.1% of CABG treated patients [2].

More recently, in the five-year outcome of the ARTS II study, there was no significant difference in survival rates between patients with complete and incomplete revascularization treated by PCI or CABG. Conversely, this study showed a significantly lower free-from-MACE survival in patients with higher SYNTAX tertiles compared to the lower and intermediate ones [14].

## 3. Assessment of Cardiovascular Risk after Revascularization

*Exercise testing *is a widely used method particularly valuable in assessing cardiovascular status after the occurrence of a cardiac event or therapeutic interventions such as PCI. It may provide useful information on symptoms and functional capacity of the patient. When it is performed after discharge it is helpful for activity counseling and/or exercise training as part of the cardiac rehabilitation program [15]. However, data obtained from two meta-analyses demonstrate that exercise testing, even if information from the electrocardiogram and symptomatic data is synthesized, is poor diagnostic for myocardial ischemia with a sensitivity of 46% and a specificity of 77% [6, 7]. The use of stress nuclear imaging increases significantly the sensitivity to 87% and the specificity to 78%, while that of stress echocardiography imaging increases the sensitivity to 63% and the specificity to 87%. The lower sensitivity of the exercise ECG compared to imaging techniques in clinical practice is worsened further by inadequate stress yielding low exercise heart rates, the use of drugs that are known to influence test results, and the extent of disease in vessels other than those dilated. Furthermore, the exercise electrocardiogram does not permit the determination of location of the ischemia, nor does it accurately assess the extent of ischemia; these factors are often crucial in the clinical decision making after-PCI. 

Although exercise testing has the advantage of widespread availability and relatively low cost, the higher test accuracy obtained by stress imaging provides greater advantages for clinical assessment of these patients. An additional advantage is that pharmacological imaging stress testing may be performed in patients who are unable to exercise or who have an uninterpretable electrocardiogram.

**Figure 1:**
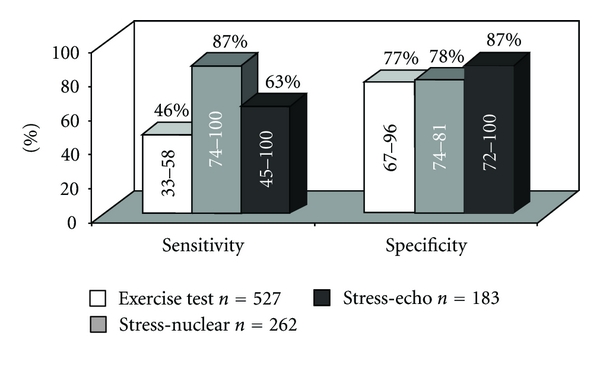
Sensitivity and specificity of functional testing for the detection of restenosis (>50% stenosis) after PCI: a meta-analysis. Adapted from Garzon and Eisenberg, Can J Cardiol 2001 [6].

**Figure 2:**
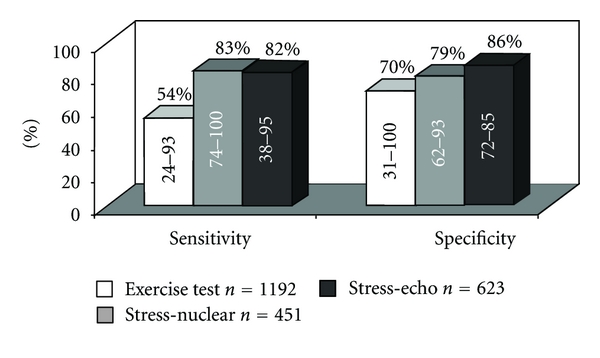
Sensitivity and specificity of functional testing for the detection of restenosis (>50% stenosis) after PCI: a meta-analysis. Adapted from Dori et al., J Intern Med 2003 [7].

Several studies [6, 7, 16–23] employing *SPECT myocardial perfusion imaging (MPI) *at different times from PCI have shown high level of sensitivity and specificity of nuclear imaging when compared to those of coronary angiography (Figures 1 and 2). The overall performance of SPECT-MPI for the detection of myocardial ischemia was 79% for both sensitivity and specificity. These values improve when MPI is performed later than 2 months after revascularization. The decreased specificity observed when MPI is performed prematurely after PCI was initially noted following PTCA [24–26] and after coronary stenting [27, 28]. Indeed, MPI may be altered as a consequence of impaired flow reserve due to an epicardial coronary stenosis or, in the absence of coronary obstruction, as a consequence of an endothelial dysfunction and medial injury at the treated site or abnormal microvascular and resistive vessel function distal to the PCI site as shown by various authors [29, 30] and by us in two editorials [31, 32]. 

Similar to MPI, echocardiography in conjunction with exercise provides useful functional assessment of coronary lesions and has a high concordance with myocardial scintigraphy [33]. Mertes and colleagues found that exercise echocardiography may predict the development of recurrent ischemia after PTCA, with a sensitivity of 83% and specificity of 85% [34]. The functional significance of a lesion may also be determined with the use of pharmacological stress [35]. For the detection of coronary restenosis, exercise [36], dipyridamole [37], and dobutamine echocardiography [38] have shown a diagnostic accuracy similar to that seen with MPI although a lower sensitivity (75% to 87%) and a slight higher specificity (84% to 95%) were found in the direct comparison with the nuclear data [36, 37]. A limitation of stress echocardiography is the inability to get acceptable quality images of left ventricle walls in about 15% of patients. This problem can be solved by using dobutamine magnetic resonance imaging. Indeed, if this exam is performed at institutions and interpreted by physicians with adequate experience and training, it is considered of major importance for coronary artery disease detection and, along with gadolinium, for viability detection to predict left ventricular function recovery [39].

## 4. Stress Imaging after Anatomical Complete or Incomplete Revascularization

Both MPI and stress echocardiography have a clear superiority with regard to specificity and predictive value for postrevascularization events [40–44]. Indeed, for MPI, these findings have been shown regardless of the method selected and include the use of 201Tl or 99mTc radiopharmaceuticals or after the use of different modes of stress imaging [45–48]. In the Angioplasty Compared to Medicine Study, 328 patients were randomized to PTCA or to medical therapy. At six months after randomization, 82% of patients underwent stress MPI and were followed for more than five years. Mortality in the PTCA group was 20% for those with a reversible defect versus 7% for those without such a defect (*P* = 0.03). By multivariate analysis, the strongest predictors of mortality were diabetes, smoking, and a reversible perfusion defect at MPI [46].

**Figure 3:**
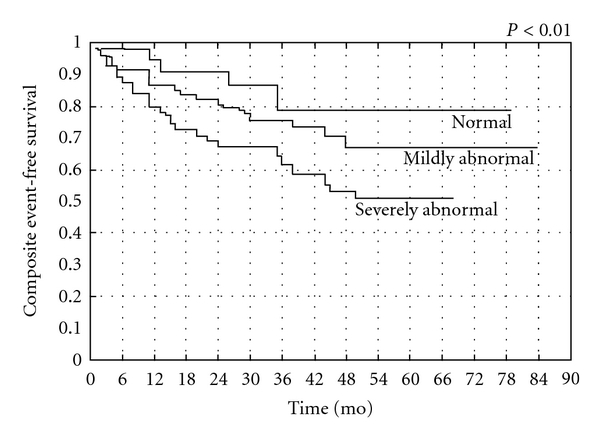
Cardiac composite event-free survival in patients with normal, mildly abnormal, or severely abnormal SPECT 99mTc-tetrofosmin scintigraphy. From Galassi et al., Am J Cardiol 2006 [8].

**Figure 4:**
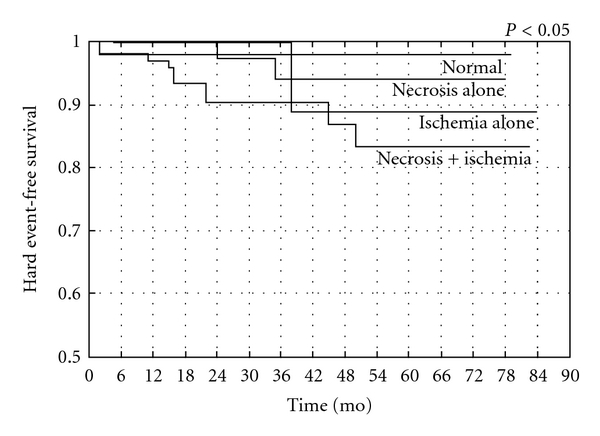
Cardiac hard event-free survival in patients with normal, mildly abnormal, or severely abnormal SPECT 99mTc-tetrofosmin scintigraphy. From Galassi et al., Am J Cardiol 2006 [8].

**Figure 5:**
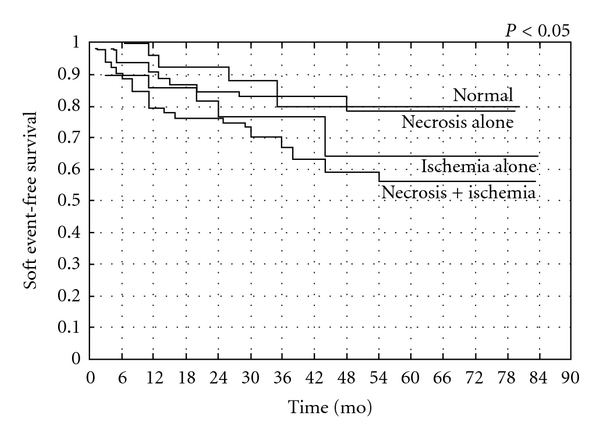
Cardiac soft event-free survival in patients with normal, mildly abnormal, or severely abnormal SPECT 99mTc-tetrofosmin scintigraphy. From Galassi et al., Am J Cardiol 2006 [8].

Similarly, the relative prognostic information derived from MPI at 1 year after revascularization for patients with multivessel coronary artery disease included in the prospective EAST Trial reveals a strong correlation between detected ischemia on thallium scintigraphy and subsequent events [45, 47]. More recently, in patients with incomplete revascularization procedures, we demonstrated that exercise SPECT MPI provides significant independent information concerning the subsequent risk of both hard and soft cardiac events, with a composite annualized event rate <2% for patients with a normal scan (Figure 3). MPI is able to provide incremental prognostic information after adjusting for clinical, angiographic, and exercise variables and is able to predict the occurrence of cardiac hard and soft events when separating patients according to the presence of myocardial ischemia and necrosis (Figures 4 and 5) [8]. The results of this study suggest that certain high-risk patients, such as those with multivessel coronary artery disease, treated by incomplete revascularization may benefit from routine MPI; this is especially true for low-risk patients as shown by Ho and colleagues who studied 211 patients between 1 and 3 years after PCI and monitored them for 7.3 years. Despite a low overall annual event rate of 1% per year, an abnormal MPI was significantly predictive of cardiac death or MI, whereas a normal MPI was associated with low risk [49].

To date, the major evidence that supports revascularization in patients with an abnormal MPI is based on the following three studies. Hachamovitch et al. examined a prospective database from a single center and classified patients on the basis of the severity of ischemia on stress MPI and examined the effect of revascularization versus medical therapy on subsequent mortality after an average followup of two years. There was no difference in mortality between medical therapy and revascularization in patients with ischemia <10% of myocardium, while patients with an ischemia >10% had lower mortality with revascularization [50]. These results were also confirmed in the nuclear substudy of the COURAGE trial, in which patients with stable angina had paired MPI studies performed at baseline and 6–18 months after randomization of optimal medical therapy versus optimal medical therapy and PCI. A reduction of ischemia >5% on MPI was associated with a 13.4% rate of the death/myocardial infarction composite event rate, whereas in those with no reduction of ischemia the composite event rate was 27.4% (more than two-fold). Indeed, PCI was more effective in achieving an ischemia reduction of >5% compared to medical therapy (33% versus 19.8%, *P* = 0.004) [51]. These two studies suggest that the benefits of revascularization over medical therapy are related rather than to a more complete anatomical revascularization, to a significant reduction of ischemia, revascularization driven.

**Figure 6:**
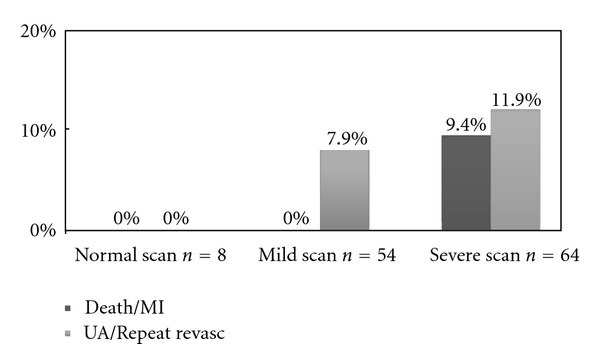
Rate of death/myocardial infarction and unstable angina/repeat revascularization in patients with normal, mildly abnormal, and severely abnormal SPECT Tc-tetrofosmin scintigraphy after a 4-year followup, in presence of CTO. MI: myocardial infarction; UA: unstable angina, From Galassi et al., J Interven Cardiol 2010 [9].

Similarly, in a recent study by our group in patients with a residual CTO in a main coronary artery left untreated after PCI who underwent follow-up stress MPI, myocardial perfusion imaging provided significant independent information concerning the subsequent risk of cardiac events. Indeed, patients with severe ischemia had significantly higher rates of death and myocardial infarction than those with either mild ischemia or no ischemia. Furthermore, by characterizing the type of defect in terms of presence of scar, ischemia only, or scar and ischemia, we observed that patients with ischemia had the worst outcomes. This study emphasizes the prognostic significance of ischemia on stress MPI even in patients with chronically occluded coronary vessels, so refocusing the attention on the difference between anatomic and functional revascularization [9]. This study, with all others from literature, underlines that it is not the anatomic severity of coronary disease which determines prognosis, rather the achievement of a “functionally” complete revascularization, in other words revascularization of all coronary arteries which serve only ischemic and viable myocardial segments [9, 13, 52, 53] (Figure 6). This is why many studies evaluating the impact of completeness of revascularization strategy based only on anatomic criteria (coronary stenosis ≥50%) have failed to demonstrate improvements in survival rates.

## 5. Conclusions

**Figure 7:**
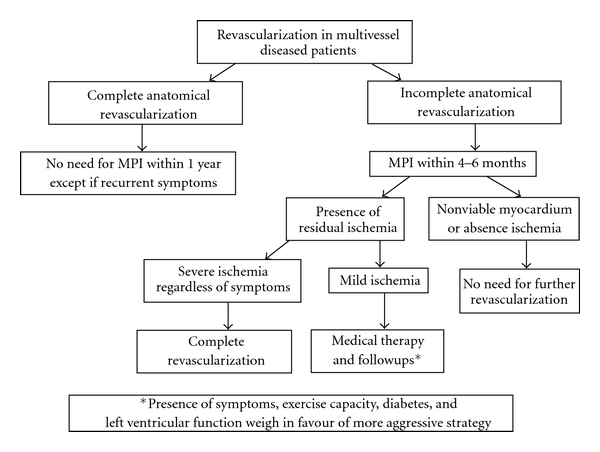
Flow chart algorithm in patients with multivessel disease. MPI: myocardial perfusion imaging.

Although complete revascularization remains a desirable goal in multivessel disease patients, incomplete revascularization strategy may be a preferred treatment strategy and a common clinical practice in many selected cases, when wisely chosen, because of its easier achievement and lower immediate risk. Among these incompletely revascularized patients, MPI is able to identify those patients with ischemic and viable myocardial regions, in which a “functionally complete revascularization” is needed to improve their outcome (Figure 7). 

##  Conflict of Interests

The authors declared that there is no conflict of interests.
